# Proteomic identification of heterogeneous nuclear ribonucleoprotein L as a novel component of SLM/Sam68 Nuclear Bodies

**DOI:** 10.1186/1471-2121-10-82

**Published:** 2009-11-13

**Authors:** Prabhakar Rajan, Caroline Dalgliesh, Cyril F Bourgeois, Monika Heiner, Kaveh Emami, Emma L Clark, Albrecht Bindereif, James Stevenin, Craig N Robson, Hing Y Leung, David J Elliott

**Affiliations:** 1 Institute of Human Genetics, Newcastle University, Central Parkway, Newcastle-upon-Tyne, NE1 3BZ, UK; 2 Beatson Institute for Cancer Research, Garscube Estate, Switchback Road, Glasgow, G61 1BD, UK; 3 IGBMC Department of Functional Genomics, Illkirch, F-67400, France; 4 INSERM U596, Illkirch, F-67400, France; 5 CNRS UMR 7104, Illkirch F-67400, France; 6 University of Strasbourg, Strasbourg, F-67000, France; 7 Institute of Biochemistry, Department of Biology and Chemistry, Justus-Liebig-University of Giessen, Heinrich-Buff-Ring 58, D-35392 Giessen, Germany; 8 North East Proteome Analysis Facility, Devonshire Building, Newcastle University, Devonshire Terrace, Newcastle-upon-Tyne, NE1 7RU, UK; 9 Northern Institute for Cancer Research, Newcastle University, Framlington Place, Newcastle-upon-Tyne, NE2 4HH, UK

## Abstract

**Background:**

Active pre-mRNA splicing occurs co-transcriptionally, and takes place throughout the nucleoplasm of eukaryotic cells. Splicing decisions are controlled by networks of nuclear RNA-binding proteins and their target sequences, sometimes in response to signalling pathways. Sam68 (Src-associated in mitosis 68 kDa) is the prototypic member of the STAR (Signal Transduction and Activation of RNA) family of RNA-binding proteins, which regulate splicing in response to signalling cascades. Nuclear Sam68 protein is concentrated within subnuclear organelles called SLM/Sam68 Nuclear Bodies (SNBs), which also contain some other splicing regulators, signalling components and nucleic acids.

**Results:**

We used proteomics to search for the major interacting protein partners of nuclear Sam68. In addition to Sam68 itself and known Sam68-associated proteins (heterogeneous nuclear ribonucleoproteins hnRNP A1, A2/B1 and G), we identified hnRNP L as a novel Sam68-interacting protein partner. hnRNP L protein was predominantly present within small nuclear protein complexes approximating to the expected size of monomers and dimers, and was quantitatively associated with nucleic acids. hnRNP L spatially co-localised with Sam68 as a novel component of SNBs and was also observed within the general nucleoplasm. Localisation within SNBs was highly specific to hnRNP L and was not shared by the closely-related hnRNP LL protein, nor any of the other Sam68-interacting proteins we identified by proteomics. The interaction between Sam68 and hnRNP L proteins was observed in a cell line which exhibits low frequency of SNBs suggesting that this association also takes place outside SNBs. Although ectopic expression of hnRNP L and Sam68 proteins independently affected splicing of *CD44 *variable exon v5 and *TJP1 *exon 20 minigenes, these proteins did not, however, co-operate with each other in splicing regulation of these target exons.

**Conclusion:**

Here we identify hnRNP L as a novel SNB component. We show that, compared with other identified Sam68-associated hnRNP proteins and hnRNP LL, this co-localisation within SNBs is specific to hnRNP L. Our data suggest that the novel Sam68-hnRNP L protein interaction may have a distinct role within SNBs.

## Background

Alternative splicing is regulated in part by a network of signalling pathways which respond to extracellular stimuli [1]. One molecule with a key role linking signalling and splicing is Sam68 (Src-associated in mitosis 68 kDa). Sam68 is the prototypic member of the STAR (Signal Transduction and Activation of RNA) family of RNA-binding proteins [2]. Sam68-dependent splicing events impact upon important cellular decisions such as choices between cell survival and cell death [3]. Sam68 has also been recently shown to play an important role in neurogenesis through splicing regulation of specific pre-mRNAs important for neural development [4]. Homozygous null Sam68 mice have pleiotropic defects in bone morphogenesis, spermatogenesis and motor coordination suggesting widespread anatomical functions for the encoded protein [5,6].

Sam68 has been reported to be associated with a number of different proteins involved in RNA processing, transcription, and cell signalling. Sam68 induces ERK (extracellular signal-regulated kinase)-mediated inclusion of *CD44 *variable exon v5 in response to Ras activation [7]. Activation of *CD44 *exon v5 splicing by Sam68 involves interactions with U2AF65 to facilitate v5 exon definition [8]. Sam68 also interacts with the splicing repressor hnRNP A1 [3] and nuclear transcriptional regulators [9,10]. The amino acid sequence of Sam68 protein contains several consensus motifs that mediate protein-protein and protein-RNA interactions in response to different stimuli [2]. In most somatic cells, Sam68 protein is exclusively nuclear, but Sam68 can also interact with cytoplasmic signalling molecules [2]. Sam68 protein has also been observed in the cytoplasm of secondary spermatocytes, where it is associated with polysomes and is involved in translational regulation [11,12].

In cancer cells, Sam68 protein exhibits a general nucleoplasmic distribution but is also concentrated within subnuclear structures called SLM/Sam68 Nuclear Bodies (SNBs) [13]. Although the exact function of SNBs is unknown, they have been shown to contain some other splicing regulators, signalling components and nucleic acids. Although Sam68 has a number of reported interacting protein partners, its major associated proteins are not yet known. In this study, we searched by proteomics for the major interacting protein partners of nuclear Sam68. Our data reveal hnRNP (heterogeneous nuclear ribonucleoprotein) L as a novel Sam68-associated nuclear protein and a novel component of SNBs.

## Results

### hnRNP L is a novel Sam68-interacting protein

**Figure 1:**
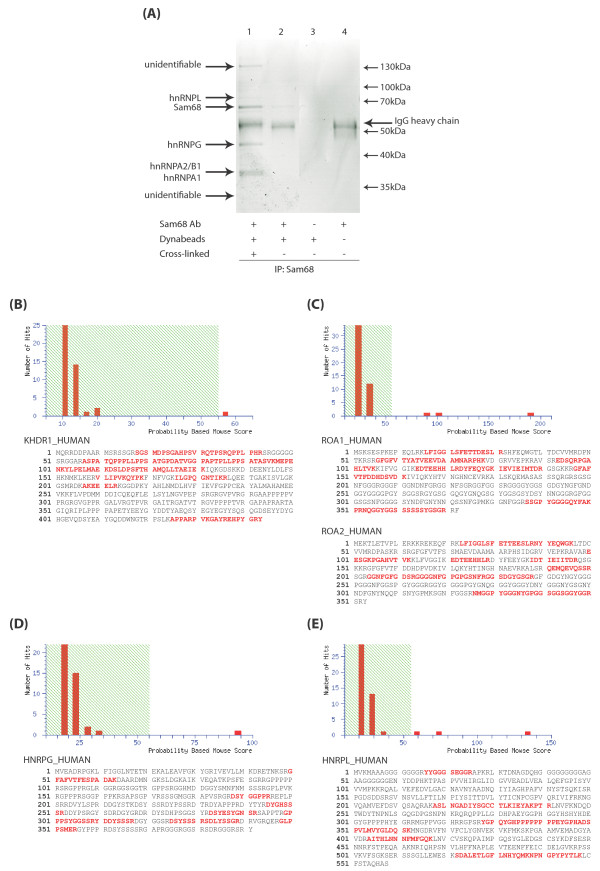
**Proteomic analysis identifies hnRNP L as a major nuclear Sam68-interacting protein**. (A) LNCaP cell nuclear extracts were subjected to immunoprecipitation (IP) using anti-Sam68 rabbit antisera. IP was carried out in the presence or absence (+/-) of antisera to Sam68, with or without (+/-) Dynabeads Protein A, and either with or without (+/-) prior cross-linking of the antisera to the Dynabeads Protein A. Immunoprecipitates were resolved by SDS-PAGE, and six bands were excised from the Coomassie-stained gel for identification by peptide mass fingerprinting, of which 4 were positively identified. (B to E) MASCOT search results for Sam68-interacting proteins: Swiss-Prot entry names KHDR1_HUMAN (B: Sam68), ROA1_HUMAN (C: hnRNP A1), ROA2_HUMAN (C: hnRNP A2/B1), HNRPG_HUMAN (D: hnRNP G), and HNRPL_HUMAN (E: hnRNP L). Histograms show the MOWSE score distributions for identified peptides, with matched peptide sequences shown in bold red. The band corresponding to histogram (C) contained a mixture of peptides matching to Swiss-Prot entry names ROA1_HUMAN and ROA2_HUMAN.

Sam68 protein is expressed at high levels in the nuclei of prostate cancer cells [10,14]. We immunoprecipitated endogenous Sam68 protein in nuclear extracts prepared from LNCaP cells. Western analysis confirmed that Sam68 protein was efficiently immunoprecipitated by rabbit antisera specific for Sam68, but not by normal rabbit IgG (Additional File 1). Further analysis of the immunoprecipitates using SDS-PAGE followed by Coomassie staining revealed a number of immunoprecipitated proteins, including a protein of ~68 kDa and a protein co-migrating with IgG heavy chain (Figure 1A; compare lanes 1 and 4), as well as a number of other distinct proteins. Peptide mass fingerprinting by MALDI-TOF (Matrix-Assisted Laser Desorption/Ionization Time Of Flight) mass spectrometry was carried out on each of the immunoprecipitated proteins. The Mascot search engine was used to match peptides with statistically significant MOWSE (Molecular Weight Search) scores mapping across full-length protein sequences obtained from the Swiss-Prot database (Figure 1B to 1E and Table 1). We identified the ~68 kDa band as Sam68 (Figure 1B), and other co-immunoprecipitated proteins as the known Sam68-interacting proteins hnRNP A1 [3] (Figure 1C), hnRNP G [15] (Figure 1D), and hnRNP A2/B1 [16] (Figure 1C).

**Table 1:** Identities of Sam68-interacting proteins.

Swiss-Prot entry name	Accession Number	Gene name	Protein ID	MOWSE Score	p-value	Peptides matched	Sequence Coverage	Nominal mass Mr	Calculated pI value
KHDR1_HUMAN	Q07666	KHDRBS1	Sam68	57	p = 0.036	12	33%	48311	8.73
ROA1_HUMAN	P09651	HNRNPA1	hnRNP A1	90	p < 0.001	10	34%	38936	9.26
ROA2_HUMAN	P22626	HNRNPA2B1	hnRNP A2/B1	100	p < 0.001	11	35%	37464	8.97
HNRPG_HUMAN	P38159	RBMX	hnRNP G	94	p < 0.001	9	20%	42306	10.06
HNRPL_HUMAN	P14866	HNRNPL	hnRNP L	134	p < 0.001	7	19%	60719	6.65

Swiss-Prot names and primary accession numbers for proteins identified by Mascot searches. *p*-values were calculated using the MOWSE algorithm. Nominal mass (*Mr*) and isoelectric point (pI) were calculated using the integer mass of the most abundant isotope of each constituent element and the individual ionizing groups in each peptide, respectively.

**Figure 2:**
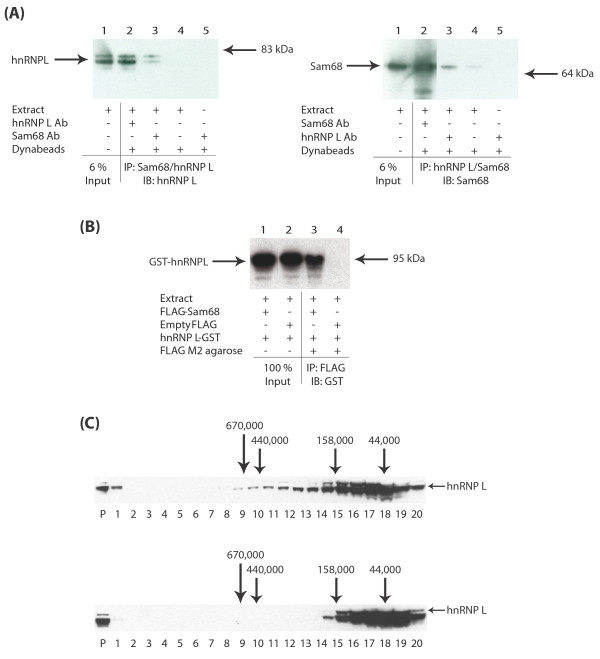
**Sam68 and hnRNP L proteins associate via protein-protein interactions and are not bridged by nucleic acids**. (A) LNCaP cell nuclear extracts were subjected to IP using either anti-Sam68 antisera or an anti-hnRNP L monoclonal antibody. Recovered material was subjected to Western analysis with antibodies specific to hnRNP L (left panel) or Sam68 (right panel). (B) HEK293 cells were transfected with expression vectors for FLAG-Sam68 or FLAG alone, and hnRNP L-GST, and subjected to IP using anti-FLAG M2 agarose. Recovered material was subjected to Western analysis with antisera to GST. (C) HEK293 cell nuclear extracts were fractionated on sucrose gradients before (upper panel) or after (lower panel) treatment with MNase to digest nucleic acids. Fraction 20 (containing low molecular weight material) was taken from the top of the gradient, and fraction 1 (containing high molecular weight material) was taken from the bottom. Pelleted material is indicated as P. The migration of individual proteins in each fraction was monitored by SDS-PAGE and Western analysis. The mobility of size markers on the gradients is shown.

In addition to the three previously known Sam68-associated proteins, we also identified hnRNP L as a novel potential Sam68-interacting protein partner (Figure 1E). By Coomassie staining, the levels of co-precipitated hnRNP A1 and hnRNP G were similar to the level of immunoprecipitated Sam68, but there was considerably less co-immunoprecipitated hnRNP L (Figure 1A, lane 1). Hence, to independently confirm the novel Sam68-hnRNP L interaction, both Sam68 and hnRNP L proteins were separately immunoprecipitated in LNCaP cell nuclear extracts using their cognate antibodies prior to Western analysis (Figure 2A, left and right panels). hnRNP L protein was efficiently immunoprecipitated by a monoclonal antibody specific for hnRNP L, and also co-immunoprecipitated by the antisera to Sam68 (Figure 2A, left panel). Likewise, Sam68 protein was efficiently immunoprecipitated by its cognate antisera, and also co-immunoreprecipitated by the anti-hnRNP L antibody (Figure 2A, right panel). Similar to previous reports [17], we observed hnRNP L to migrate as a doublet, and both of these isoforms interacted with Sam68 (Figure 2, left panel). No or very weak immunoprecipitation was observed in the absence of either antibodies (Figure 2A, both panels, lane 4).

To further verify the novel Sam68-hnRNP L detected interaction, HEK293 cells were transiently transfected with expression constructs encoding FLAG-tagged Sam68 and GST-tagged hnRNP L proteins. Anti-FLAG M2 agarose was used to immunoprecipitate FLAG-tagged proteins from HEK293 cells, and co-immunoprecipitated GST fusion proteins were detected using antisera specific to GST. Consistent with the observed interactions of endogenous proteins, hnRNP L-GST was efficiently co-immunoprecipitated with Sam68-FLAG (Figure 2B, compare lanes 1 and 3), but could not be co-immunoprecipitated in cells transfected with an empty FLAG vector (Figure 2B, compare lanes 2 and 4).

Note that all immunoprecipitations of both endogenous (Figure 2A) and FLAG-tagged proteins (Figure 2B) were carried out in the presence of Benzonase nuclease to ensure that the detected associations were mediated by protein-protein interactions and not bridged by nucleic acids.

### Sam68 and hnRNP L are both present in small protein complexes which are quantitatively associated with nucleic acids

Sam68 is quantitatively associated with small nuclear protein complexes of a size compatible with either monomers or dimers [18]. In order to investigate and compare the size ranges of any endogenous nuclear complexes containing hnRNP L protein, we analysed velocity gradient fractions of HEK293 cell nuclear extracts prepared either without pretreatment or after prior micrococcal nuclease (MNase) treatment to eliminate any nucleic acid dependent complexes (Figures 2C and [18]). Similarly to previously published data for Sam68, following MNase treatment, hnRNP L (monomeric molecular weight ~70 kDa) protein was mainly present in small stable protein complexes (of less than 158 kDa) corresponding to the expected size of monomeric or dimeric molecular complexes (Figure 2C, upper panel). Also like Sam68, hnRNP L protein was quantitatively associated with nucleic acids since it migrated with much larger complexes without prior MNase digestion (Figure 2C, lower panel).

### hnRNP L uniquely co-localises with Sam68 as a novel component of SNBs

**Figure 3:**
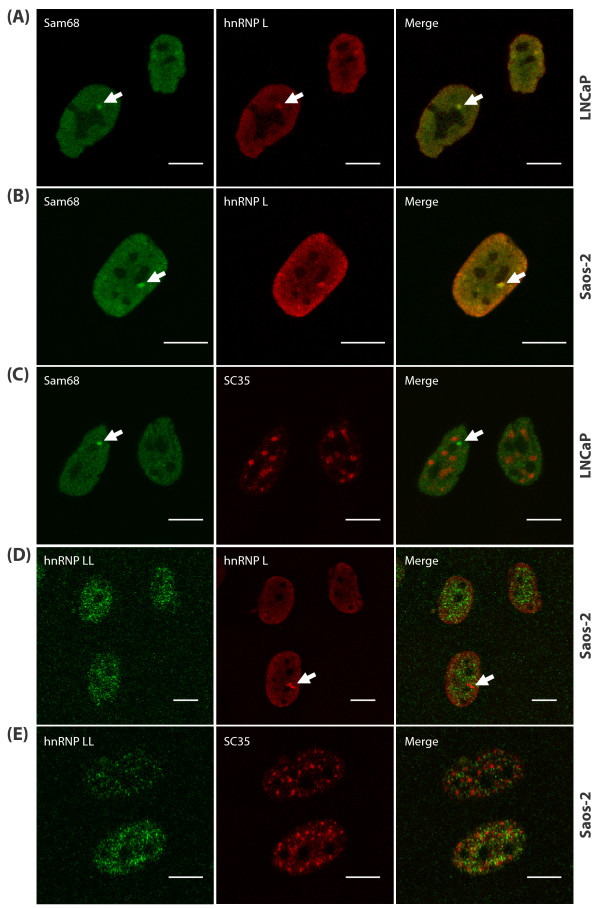
**Endogenous hnRNP L protein co-localises with Sam68 within SNBs**. Representative images of LNCaP and Saos-2 cells captured by confocal laser scanning microscopy using indirect immunofluorescence and antibodies specific to Sam68, hnRNP L, hnRNP LL and SC35. (A and B) Sam68 co-localises with hnRNP L to SNBs (arrowed), but not with SC35 to splicing speckles (C). (D) hnRNP LL exhibits a different subnuclear localisation to hnRNP L, which is again distinct from SC35-containing splicing speckles (E), and does not co-localise to SNBs (arrowed). (Bar = 10 μm).

Within the nuclei of cancer cells, Sam68 protein exhibits a general nucleoplasmic distribution but is also concentrated within distinct peri-nucleolar structures called SNBs [13]. We compared the localization of Sam68 and hnRNP L proteins in cancer cell lines using indirect immunofluorescence (representative images are shown in Figure 3A and 3B, and Additional File 2). Confocal microscopy demonstrated that the endogenous Sam68 and hnRNP L proteins directly overlapped within SNBs in 100% of all cells examined. In addition to SNBs, further large pools of hnRNP L and Sam68 proteins were seen in the general nucleoplasm, and in the case of hnRNP L, towards the nuclear periphery in Saos-2 cells (the position of representative SNBs are indicated by arrows in Figures 3A, B and Additional File 2: note also a large population of both Sam68 and hnRNP L proteins outside SNBs). In parallel experiments, using the same combination of secondary antisera, no co-localisation was observed between Sam68 protein (identified using specific rabbit antisera) and SC35-containing splicing speckles (identified using a specific mouse monoclonal antibody) (Figure 3C). The distribution of Sam68 and hnRNP L proteins was examined in three different cell lines (LNCaP, Saos-2 and HeLa) and found to be very similar suggesting that the hnRNP L association with SNBs may be ubiquitous (Figure 3A, B, and Additional File 2). Furthermore, the other identified Sam68-associated proteins (hnRNP A1, A2/B1, and G) identified in our proteomic screen exhibited diffuse subnuclear distributions and did not localise within SNBs (Additional File 2 and [19]).

Only a small number of molecules have been identified as SNB components, and each of these share a role in cell signalling and/or RNA processing. hnRNP L has a closely-related homolog within the cell called hnRNP LL (hnRNP L-like), which also regulates signal-dependent splicing. We carried out further experiments to test if hnRNP LL protein would be also found within SNBs. Surprisingly, these experiments indicated that hnRNP LL protein exhibits a nucleoplasmic distribution outside SNBs (Figure 3D and Additional File 2). The observed hnRNP LL distribution was punctate and somewhat reminiscent to that of splicing speckles. We compared the localisation of hnRNP LL protein with SC35, but found that the speckled distribution of hnRNP LL is in fact distinct from the SC35-containing splicing speckles (Figure 3E).

**Figure 4:**
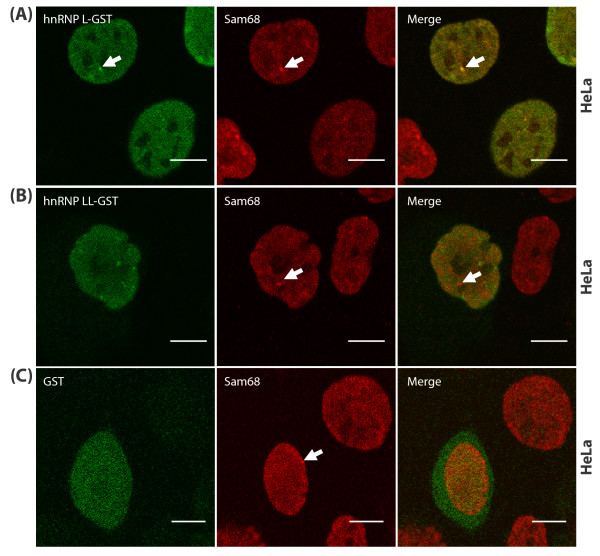
**An ectopically-expressed hnRNP L fusion protein localises within SNBs**. Representative indirect immunofluorescence images of HeLa cells transfected with vectors encoding hnRNP L-GST (A), hnRNP LL-GST (B), or GST alone (C), and captured by confocal laser scanning microscopy using indirect immunofluorescence and antibodies to Sam68 and GST. (A) Direct co-localisation within SNBs of endogenous Sam68 and ectopically-expressed hnRNP L-GST fusion protein (arrowed). (B) No co-localisation within SNBs was observed between endogenous Sam68 and the ectopically-expressed hnRNP LL-GST fusion protein. (C) GST protein was generally distributed within the nucleus and cytoplasm and showed no subnuclear localisation within SNBs, (Bar = 10 μm).

To verify these localization findings, HeLa cells were transiently transfected with expression vectors encoding hnRNP L-GST, hnRNP LL-GST, or GST alone. We then detected the localisation of the ectopically expressed proteins using antisera to detect the GST tag, and compared this distribution with that of endogenous Sam68 protein (Figure 4). Consistent with the data described above, hnRNP L-GST but not hnRNP LL-GST, was found within SNBs (Figure 4A and 4B). The GST moiety had no effect on protein localization, since GST alone was detected both within the nuclei and cytoplasm of transfected cells, and not within SNBs (Figure 4C).

### Sam68 and hnRNP L proteins associate in a cell line which exhibits a low frequency of SNBs

**Figure 5:**
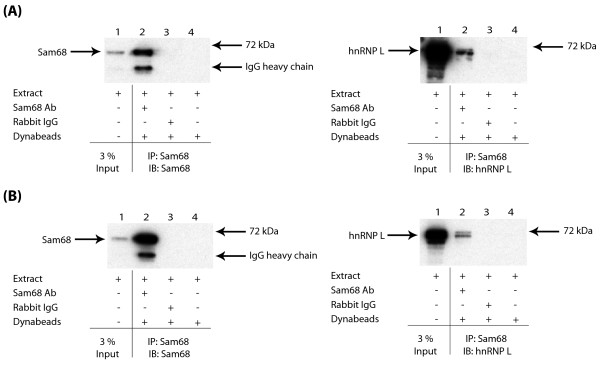
**Sam68 and hnRNP L proteins associate in a cell line which exhibits a low frequency of SNBs**. (A) LNCaP and (B) NIH3T3 whole cell extracts were subjected to IP using either anti-Sam68 antisera or normal rabbit IgG. Recovered material was subjected to Western analysis with antibodies specific to Sam68 (left hand panels) or hnRNP L (right hand panels).

The frequency of cells in a population containing SNBs is cell line-dependent. To test whether the association of Sam68 and hnRNP L was dependent on the presence of SNBs, we performed side-by-side immunoprecipitations in whole cell extracts obtained from LNCaP and NIH3T3 cells (only ~5.5% of NIH3T3 cells have SNBs) [13]. Sam68 protein was efficiently immunoprecipitated by its cognate antisera from both these cell lines (Figure 5A and 5B, left panels). Consistent with the interaction of Sam68 and hnRNP L also occurring outside of SNBs, virtually equal levels of hnRNP L protein was co-immunoprecipitated by the antisera to Sam68 from NIH3T3 cells as LNCaP cells (Figure 5A and 5B, right panels). Notice that neither Sam68 nor hnRNP L proteins were immunoprecipitated by normal rabbit IgG or beads alone. All immunoprecipitations were carried out in the presence of Benzonase nuclease to confirm that the detected associations were mediated by protein-protein interactions and not bridged by nucleic acids.

### The Sam68-hnRNP L protein interaction does not significantly impact on splicing regulation of two known target exons

The above experiments indicate that Sam68 and hnRNP L proteins interact and are both present within SNBs. Although SNBs contain some splicing regulators and RNA, they do not detectably contain key spliceosome components [13]. Hence SNBs are not thought to be sites for active pre-mRNA splicing, which takes place in the general nucleoplasm [20]. Both Sam68 and hnRNP L proteins are independently implicated in the regulation of distinct alternative splicing events during which exon inclusion can be either activated or repressed depending on the target transcript [4,21]. Although active splicing of Sam68 and hnRNP L target pre-mRNAs is unlikely to take place within SNBs, we also detected significant nucleoplasmic populations of both Sam68 and hnRNP L proteins outside SNBs, and protein interactions between Sam68 and hnRNP L occurred equally well in a cell line which exhibits a low frequency of SNBs. These observations raise the question as to whether the Sam68-hnRNP L interaction might have a co-operative effect on pre-mRNA splicing in the general nucleoplasm.

**Figure 6:**
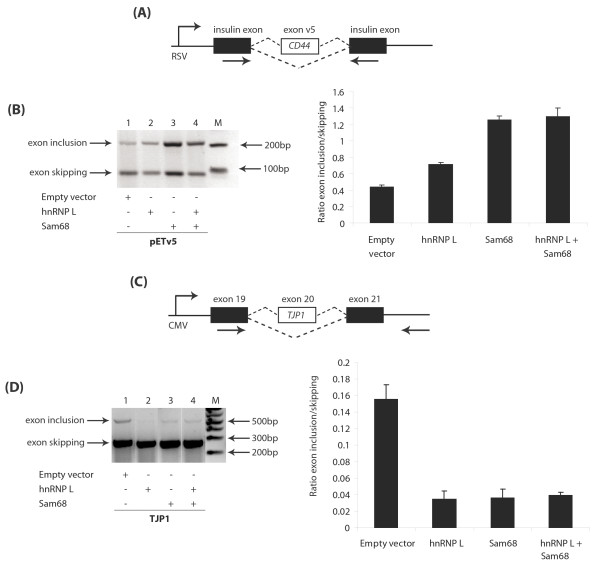
**The Sam68-hnRNP L protein interaction does not significantly impact on splicing regulation of two known target exons**. (A) Minigene pETv5 contains the *CD44 *variable exon v5 cloned downstream of a constitutively-active RSV promoter. (C) Minigene pcDNA3-*TJP1*-WT contains *TJP1 *exon 20 downstream of a constitutively-active CMV promoter. (A and C) Splicing events are shown by broken lines, and arrows show the location of primers for RT-PCR. (B) HEK293 cells were transfected with the pETv5 minigene (150 ng), and expression vectors for hnRNP L-GST or GFP-Sam68 (500 ng). (D) HEK293 cells were transfected with pcDNA3-*TJP1*-WT (150 ng), and expression vectors for hnRNP L-GST or GFP-Sam68 (500 ng). (B and D) Gel images shown are representative of at least three independent experiments, from which densitometric assessment of RT-PCR product (lower panels) was performed to obtain means +/- standard error which are plotted in the bar chart. The lane marked M shows migration of the 1 Kb plus DNA ladder (Invitrogen).

Since ectopically expressed Sam68 protein stimulates splicing inclusion of *CD44 *variable exon v5 [7], and *TJP1 *exon 20 inclusion is repressed by hnRNP L [21], we examined the effect of both Sam68 and hnRNP L protein expression on these known target exons (Figure 6). We firstly monitored splicing in HEK293 cells transfected with a minigene containing *CD44 *variable exon v5 together with expression constructs encoding Sam68, hnRNP L or both (Figure 6A). Ectopic expression of hnRNP L protein stimulated exon v5 inclusion albeit with a lower potency than Sam68 (Figure 6B, compare lane 1 with lanes 2 and 3). We carried out similar experiments with a minigene containing *TJP1 *exon 20 (Figure 6C). Due to the low levels of expression of *TJP1 *exon 20, it was not easy to draw clear conclusions from this experiment, however ectopic expression of either hnRNP L or Sam68 proteins alone appeared to repress exon 20 inclusion (Figure 6D, compare lane 1 with lanes 2 and 3). Together, these experiments indicate that both Sam68 and hnRNP L proteins can regulate the same pre-mRNA splicing events in the same direction, with hnRNP L activating *CD44 *exon v5 inclusion like Sam68, and Sam68 repressing *TJP1 *exon 20 inclusion like hnRNP L. Importantly in either case, however, co-expression of hnRNP L and Sam68 proteins did not have a significant additive effect on either *TJP1 *exon 20 skipping or *CD44 *exon v5 inclusion (Figures 6B and 6D, compare lanes 2, 3 and 4). Hence it seems unlikely that the general nucleoplasmic populations of hnRNP L and Sam68 proteins co-operate in splicing control of these shared target exons, despite their observed interaction, localisations, and ability to regulate these target exons by themselves.

## Discussion

Although Sam68 has been associated with a number of proteins, our proteomics screen identified hnRNP A1, hnRNP A2/B1, hnRNP G and hnRNP L as its four major nuclear interacting protein partners in LNCaP cells. The stoichiometry of the Sam68-associated proteins recovered by immunoprecipitation, their spatial localisations, and analysis of endogenous complexes using velocity gradient centrifugation analysis [18] are most consistent with a number of distinct small Sam68-containing protein complexes (predominantly monomeric and dimeric-sized), rather than a single large complex containing each of the identified Sam68-interacting proteins. Like Sam68 and the Sam68-interactor hnRNP G, but not hnRNP A1, hnRNP L protein was quantitatively associated with nucleic acids since it migrated with much larger complexes without prior MNase digestion before velocity gradient centrifugation.

hnRNP L protein has previously been described in discrete perinucleolar structures [17], which we here now identify as SNBs. Although the exact functions of SNBs are not fully understood, they are known to contain other STAR proteins (SLM-1 and SLM-2) [13], the splicing factor YT521B [22], Scaffold Attachment Factors SAFB1 and SAFB2 [18] and the protein kinase BRK/Sik (Breast Tumour Kinase/Src-related Intestinal Kinase) [23]. SNBs also contain nucleic acids including RNA, and on heat-shock additionally recruit hnRNP A1 and other splicing factors including arginine-serine-rich (SR) proteins [24]. Like Sam68, hnRNP L is implicated in coupling signaling and splicing: hnRNP L regulates signal-dependent splicing of *CD45 *exon v4 [25] and *STREX *(stress axis-regulated exon) [26]. Despite the spatial and physical associations of Sam68 and hnRNP L detected in our study, Sam68 and hnRNP L proteins did not either co-operate or antagonize each others' splicing activity on target exons. In contrast, the generally nucleoplasmic Sam68-associated protein hnRNP G potently inhibited Sam68-mediated splicing of *CD44 *exon v5 ([27] and Additional File 3). Our protein interaction analyses indicated that the interaction between Sam68 and hnRNP L is independent of nucleic acids. Using a directed yeast two-hybrid system, a direct protein-protein interaction has been demonstrated between the STAR-family protein rSLM-2 and hnRNP L [27], suggesting, by analogy, that the Sam68-hnRNP L interaction may also be direct. However, we were unable to detect such an interaction by directed yeast two hybrid analysis (unpublished observations).

Amongst all the Sam68-associated proteins we investigated in this study, the subnuclear distribution of hnRNP L protein is specifically within SNBs. Consistent with the known nuclear functions of hnRNP L, SNBs have been suggested as potential nuclear sites for the regulation of RNA processing by signalling pathways. However, we found that the homologous hnRNP LL protein which is also implicated in signal-dependent *CD45 *splicing [28-30], did not localize to SNBs. Hence SNBs are not a default location for all nuclear RNA-binding proteins involved in signaling and RNA processing. hnRNP LL protein lacks some proline- and glycine-rich regions present within the hnRNP L amino acid sequence, and by analogy with Sam68, it is possible that these motifs are required for interactions with signaling molecules and SNB localization. The distinct nuclear localization we observe for hnRNP LL may suggest somewhat diverged functions and so provide some explanation for the evolutionary maintenance of both hnRNP L and LL proteins within cells.

## Conclusion

We have identified hnRNP L as a novel Sam68-interacting protein partner and component of SNBs. Despite hnRNP L and Sam68 proteins also localizing to the general nucleoplasm, where pre-mRNA splicing occurs, we did not observe a role for the novel Sam68-hnRNP L protein interaction on splicing of known target exons for these RNA-binding proteins. The spatial localization of hnRNP L protein within SNBs further implicates a role for these organelles in coupling signaling to RNA processing.

## Methods

### Cell Culture and DNA transfection

LNCaP cells were cultured in RPMI-1640 (PAA Laboratories) supplemented with 10% foetal calf serum (FCS) (PAA Laboratories). HEK293, HeLa, NIH3T3, and Saos-2, and cells were cultured in Dulbecco's Modified Eagle Media (D-MEM) with glutamax-1 (PAA Laboratories) with 10% FCS. Plasmid transfections were performed as previously described [18] using Genejammer (Stratagene) according to manufacturer's instructions.

### Antibodies and plasmids

The following antibodies were used: normal rabbit IgG sc-2027 and anti-Sam68 sc-333 rabbit antisera (Santa Cruz Biotechnology); anti-hnRNP A1 9H10, anti-hnRNP L 4D11, anti-SC35 SC-35, and anti-β-actin AC15 mouse monoclonal antibodies (Sigma); anti-hnRNP A2/B1 DP3B2 mouse monoclonal antibody (Abcam); anti-hnRNP LL rabbit antisera (Aviva Systems Biology); anti-GST (glutathione *S*-transferase) goat antisera (GE Healthcare).

The following plasmids have been described previously: FLAG-Sam68 [10]; pGFP3-Sam68 [31]; pETv5 [32] (from Stefan Stamm, University of Kentucky, USA). The following plasmids were made using standard cloning procedures, the details of which are available on request: pcDNA-GST, pcDNA-hnRNP L-GST, and pcDNA-hnRNP LL-GST encode full-length GST, hnRNP L [33], and hnRNP LL with a C-terminal GST tag, respectively. pcDNA-hnRNP G encodes full-length hnRNP G. pcDNA3-*TJP1*-wWT contains exon 19, 20 and 21 of *TJP1 *with intervening intronic sequence.

### Peptide mass fingerprinting and mass spectrometry (MS)

Immunoprecipitated proteins were resolved on NuPAGE Novex 10% Bis-Tris gels (Invitrogen) and stained with Coomassie blue (GE Healthcare). Excised bands were digested with trypsin, and identified by peptide mass fingerprinting. Mass spectra were obtained using the Ultraflex II MALDI-TOF (matrix-assisted laser desorption/ionization time-of-flight) mass spectrometer (Bruker Daltonics) in standard MALDI-TOF mass spectrometry (MS) mode. The peak lists were searched against the non-redundant Swiss-Prot protein sequence database using the Mascot [34] search engine version 2.2 (Matrix Science). Probability-based MOWSE (Molecular Weight Search) [35] scores greater than 55 were considered as significant (p < 0.05).

### Immunoprecipitation

LNCaP cell nuclear extracts were obtained using the CelLytic NuCLEAR Extraction Kit (Sigma) according to manufacturer's instructions. Benzonase nuclease (Sigma) was used at 100 units/ml where indicated. Immunoprecipitations were performed using Dynabeads Protein A (Invitrogen) or anti-FLAG M2 agarose (Sigma) according to manufacturers' instructions. Where indicated, antibodies were cross-linked to Dynabeads Protein A according to manufacturer's instructions. Recovered material was resolved by SDS-PAGE and subjected to Western analysis as previously described [36].

### Velocity gradient centrifugation

Sucrose gradients were run using HEK293 cell nuclear extract, pre-treated or not with micrococcal nuclease (MNase), and recovered material resolved by SDS-PAGE and subjected to Western analysis as previously described [18].

### Indirect immunofluorescence microscopy

LNCaP, HeLa, and Saos-2 cells were grown and transfected on glass coverslips (VWR International), and stained with primary and secondary antibodies as previously described [18]. All images were captured using the LSM510 (Zeiss) or SP2 MP (Leica) confocal microscopes and associated software.

### Minigene splicing assays

HEK293 cells were grown in 6-well plates (Asahi Techno Glass), and transfected with DNA as detailed in figure legends. RNA was extracted using TRIzol (Invitrogen) and RT-PCR was performed using the One-Step RT-PCR kit (Qiagen) as previously described [31]. Densitometric band quantification was performed as previously described [37]. All experiments shown are the mean of at least three independent experiments ± standard error.

## Authors' contributions

PR conceived of the study, designed and performed experiments, analysed data and drafted the manuscript. CD designed and performed experiments. CB designed and performed experiments. MH contributed reagents and expertise. KE carried out the mass spectrometry. EC contributed reagents and expertise. AB contributed reagents, expertise, and to the preparation of the manuscript. JS designed experiments, contributed reagents, expertise and to the preparation of the manuscript. CR contributed reagents, expertise and to the preparation of the manuscript. HYL conceived of the study, contributed expertise and to the preparation of the manuscript. DJE conceived of the study, contributed reagents and expertise, and drafted the manuscript. All authors read and approved the final manuscript.

## Supplementary Material

Additional file 1 **Sam68 is efficiently immunoprecipitated by its cognate antisera**. LNCaP cell nuclear extracts were subjected to immunoprecipitation (IP) using anti-Sam68 rabbit antisera. IP was carried out in the presence or absence (+/-) of antisera to Sam68 or normal rabbit IgG (negative control), with or without (+/-) Dynabeads Protein A, and either with or without (+/-) prior cross-linking of the antisera to Sam68 to the Dynabeads Protein A. Recovered material was subjected to Western analysis with the antisera to Sam68. Western analysis confirmed that Sam68 protein was efficiently immunoprecipitated by its cognate antisera, and that this was most efficient if the antisera were cross-linked to the Dynabeads Protein A prior to incubation with nuclear extracts (compare lanes 3 and 4). Sam68 protein was not pulled down by Dynabeads Protein A alone (lane 2). Click here for file

Additional file 2 **hnRNPs A1 or A2/B1 do not localise within SNBs.** (A to D) Representative indirect immunofluorescence images of HeLa cells captured by confocal laser scanning microscopy using antibodies to hnRNPs A1, A2/B1, hnRNP L, hnRNP LL, and Sam68. hnRNPs A1 (A) and A2/B2 (B) exhibit a diffuse nucleoplasmic distribution but do not co-localise with Sam68 to SNBs (arrowed). (C) Sam68 co-localises with hnRNP L to SNBs (arrowed). (D) hnRNP LL exhibits a different subnuclear localisation to hnRNP L and does not co-localise to SNBs. (Bar = 10 μm). Click here for file

Additional file 3 **hnRNP G represses Sam68-mediated CD44 variable exon v5 inclusion.** (A) Minigene pETv5 contains the CD44 variable exon v5 cloned downstream of a constitutively-active RSV promoter. Splicing events are shown by broken lines, and arrows show the location of primers for RT-PCR. (B) HEK293 cells were transfected with the pETv5 minigene (150 ng), and expression vectors for hnRNP G or GFP-Sam68 (500 ng). The gel images are representative of at least three independent experiments, from which densitometric assessment of RT-PCR product was performed to obtain means +/- standard error (shown in bar chart). The lane marked M shows the migration of the 1 Kb plus DNA ladder (Invitrogen). Ectopic expression of hnRNP G protein repressed CD44 variable exon v5 inclusion both in the presence and absence of ectopic Sam68 (compare lane 1 with lane 3, and lane 2 with lane 4). Click here for file
