# Supplementary figures and images for: Proteomic identification of heterogeneous nuclear ribonucleoprotein L as a novel component of SLM/Sam68 Nuclear Bodies
Source: BMC Cell Biol. 2009 Nov 13;10:82. doi: 10.1186/1471-2121-10-82 (PMC2784748; doi:10.1186/1471-2121-10-82)

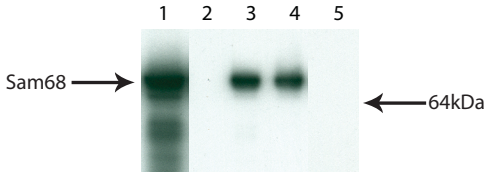

100 %  
Input

IP: Sam68  
IB: Sam68

Supplement: Additional file 1 — Sam68 is efficiently immunoprecipitated by its cognate antisera. LNCaP cell nuclear extracts were subjected to immunoprecipitation (IP) using anti-Sam68 rabbit antisera. IP was carried out in the presence or absence (+/-) of antisera to Sam68 or normal rabbit IgG (negative control), with or without (+/-) Dynabeads Protein A, and either with or without (+/-) prior cross-linking of the antisera to Sam68 to the Dynabeads Protein A. Recovered material was subjected to Western analysis with the antisera to Sam68. Western analysis confirmed that Sam68 protein was efficiently immunoprecipitated by its cognate antisera, and that this was most efficient if the antisera were cross-linked to the Dynabeads Protein A prior to incubation with nuclear extracts (compare lanes 3 and 4). Sam68 protein was not pulled down by Dynabeads Protein A alone (lane 2). [file 1471-2121-10-82-S1.pdf]

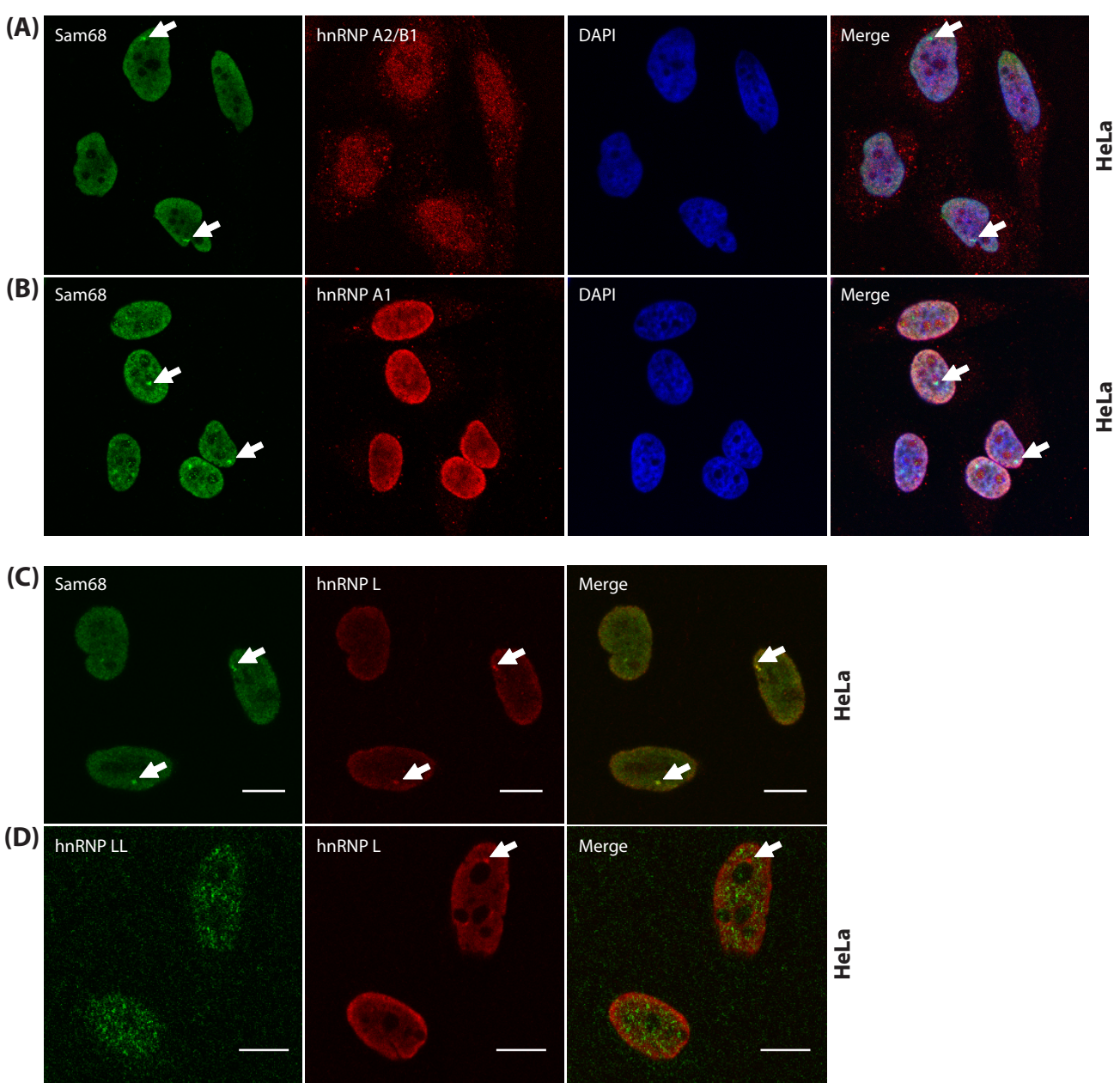

Supplement: Additional file 2 — hnRNPs A1 or A2/B1 do not localise within SNBs. (A to D) Representative indirect immunofluorescence images of HeLa cells captured by confocal laser scanning microscopy using antibodies to hnRNPs A1, A2/B1, hnRNP L, hnRNP LL, and Sam68. hnRNPs A1 (A) and A2/B2 (B) exhibit a diffuse nucleoplasmic distribution but do not co-localise with Sam68 to SNBs (arrowed). (C) Sam68 co-localises with hnRNP L to SNBs (arrowed). (D) hnRNP LL exhibits a different subnuclear localisation to hnRNP L and does not co-localise to SNBs. (Bar = 10 μm). [file 1471-2121-10-82-S2.pdf]

**(A)**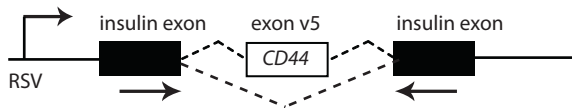**(B)**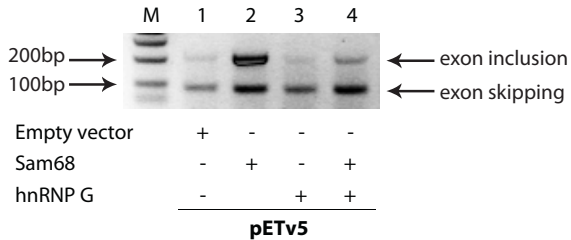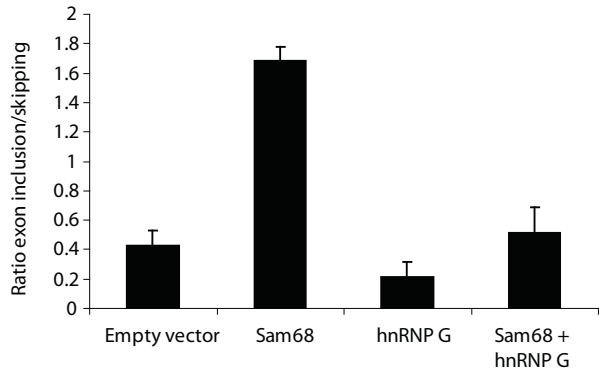

Supplement: Additional file 3 — hnRNP G represses Sam68-mediated CD44 variable exon v5 inclusion. (A) Minigene pETv5 contains the CD44 variable exon v5 cloned downstream of a constitutively-active RSV promoter. Splicing events are shown by broken lines, and arrows show the location of primers for RT-PCR. (B) HEK293 cells were transfected with the pETv5 minigene (150 ng), and expression vectors for hnRNP G or GFP-Sam68 (500 ng). The gel images are representative of at least three independent experiments, from which densitometric assessment of RT-PCR product was performed to obtain means +/- standard error (shown in bar chart). The lane marked M shows the migration of the 1 Kb plus DNA ladder (Invitrogen). Ectopic expression of hnRNP G protein repressed CD44 variable exon v5 inclusion both in the presence and absence of ectopic Sam68 (compare lane 1 with lane 3, and lane 2 with lane 4). [file 1471-2121-10-82-S3.pdf]
